# Complement Inhibition Promotes Endogenous Neurogenesis and Sustained Anti-Inflammatory Neuroprotection following Reperfused Stroke

**DOI:** 10.1371/journal.pone.0038664

**Published:** 2012-06-26

**Authors:** Andrew F. Ducruet, Brad E. Zacharia, Sergey A. Sosunov, Paul R. Gigante, Mason L. Yeh, Justin W. Gorski, Marc L. Otten, Richard Y. Hwang, Peter A. DeRosa, Zachary L. Hickman, Paulina Sergot, E. Sander Connolly

**Affiliations:** Department of Neurological Surgery, Columbia University, New York, New York, United States of America; Hertie Institute for Clinical Brain Research, University of Tuebingen, Germany

## Abstract

**Background and Purpose:**

The restoration of blood-flow following cerebral ischemia incites a series of deleterious cascades that exacerbate neuronal injury. Pharmacologic inhibition of the C3a-receptor ameliorates cerebral injury by attenuating post-ischemic inflammation. Recent reports also implicate C3a in the modulation of tissue repair, suggesting that complement may influence both injury and recovery at later post-ischemic time-points.

**Methods:**

To evaluate the effect of C3a-receptor antagonism on post-ischemic neurogenesis and neurological outcome in the subacute period of stroke, transient focal cerebral ischemia was induced in adult male C57BL/6 mice treated with multiple regimens of a C3a receptor antagonist (C3aRA).

**Results:**

Low-dose C3aRA administration during the acute phase of stroke promotes neuroblast proliferation in the subventricular zone at 7 days. Additionally, the C3a receptor is expressed on T-lymphocytes within the ischemic territory at 7 days, and this cellular infiltrate is abrogated by C3aRA administration. Finally, C3aRA treatment confers robust histologic and functional neuroprotection at this delayed time-point.

**Conclusions:**

Targeted complement inhibition through low-dose antagonism of the C3a receptor promotes post-ischemic neuroblast proliferation in the SVZ. Furthermore, C3aRA administration suppresses T-lymphocyte infiltration and improves delayed functional and histologic outcome following reperfused stroke. Post-ischemic complement activation may be pharmacologically manipulated to yield an effective therapy for stroke.

## Introduction

The complement cascade is a component of the innate immune system that plays a critical role in post-ischemic inflammation [1]. Of the numerous peptides generated though sequential complement cleavage, the anaphylatoxins, C3a and C5a, are among the most potent of all known inflammatory mediators. By binding to their cognate receptors, the C3a receptor (C3aR) and C5a receptor (C5aR), these peptides mediate their inflammatory effects across a variety of pathologic settings by promoting vascular permeability, leukocyte activation, and chemotaxis [2], [3].

Modulation of complement in animal models of stroke has proven effective in suppressing post-ischemic inflammation [4], [5], [6], [7]. Recently, a role for complement activation in tissue regeneration has been proposed [8], [9], [10]. More specifically, complement may regulate the process of endogenous neurogenesis, as neural progenitor cells and immature neurons have been reported to express both C3aR and C5aR [11]. In this work, C3- and C3aR-deficient mice exhibit impaired basal neurogenesis in the subventricular zone (SVZ). These data argue that long-term administration of anti-complement therapeutics may actually impair neurorestorative processes in the brain, potentially exacerbating post-ischemic neurological injury.

Given the potentially overlapping roles for complement in both early tissue injury and delayed regeneration, we hypothesized that the temporal distinction between the processes of injury and repair would allow for the rational design of anti-complement strategies that optimize outcome by inhibiting inflammation without impairing recovery. We thus set out to evaluate multiple regimens of C3aRA administration while rigorously assessing functional and histological outcome, including neurogenesis in the SVZ, at 7 days following reperfused stroke.

## Materials and Methods

### Ethics Statement

All experimental methods were approved by the Columbia University Institutional Animal Care & Use Committee (Protocol #: AC-AAAC2213). Adult male C57BL/6 mice (8–12 weeks, 23–26 g) obtained from Jackson Laboratories (Bar Harbor, ME, USA), were housed in certified barrier facilities with free access to food and water. Surgical procedures were performed under isoflurane anesthesia. Injections, functional testing and live handling of mice were done with all efforts to minimize distress.

**Figure 1:**
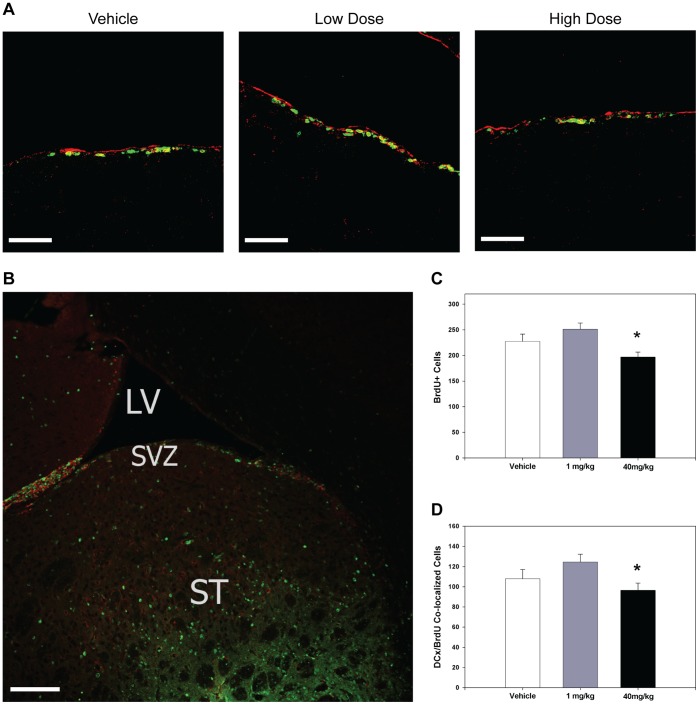
High-dose C3aRA suppresses basal neurogenesis in the SVZ relative to a low-dose regimen. Basal neurogenesis in the SVZ depicted by representative immunostaining of the SVZ of animals treated for 10 days with vehicle, low dose (1 mg/kg/day) and high dose (40 mg/kg/day) of C3aRA. BrdU staining is represented in green, DCx staining in red, and co-localized cells appear yellow. Images are representative of findings from at least 4 animals per experiment. Scale bar = 120 µm (**A**). Representative low-power immunostain of a coronal section depicting the anatomic relationships between the SVZ, the striatum (ST) and lateral ventricle (LV). BrdU staining is represented in green and DCx staining in red. Scale bar = 120 µm (**B**). Decreased BrdU+ cells (p = 0.01*) were observed in the SVZ in the high-dose cohort (n = 10 per cohort) relative to vehicle (**C**). BrdU and DCX co-localized cell counts in the SVZ were also suppressed (p = 0.05*) with high-dose treatment relative to vehicle (n = 10 per cohort) (**D**).

**Figure 2:**
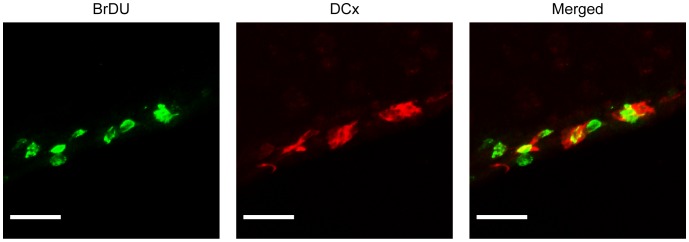
Reperfused stroke stimulates the proliferation of migrating neuroblasts in the SVZ at 7 days post-ischemia. Representative high-power split-channel immunostain of the SVZ at 7 days post-reperfusion for BrdU (green) and DCx (red), demonstrating robust co-localization (yellow) of these proteins. Scale bar = 40 µm.

### Mice

Mice utilized for the infarct volume studies, neurological function evaluation, and histological analyses were randomized to receive intraperitoneal injections of either C3aRA (1 mg/kg) (SB290157; Calbiochem, Darmstadt, Germany) diluted in phosphate-buffered saline (PBS) and dimethylsulfoxide(DMSO)(1.16%, v/v), or an equal volume of vehicle (PBS/DMSO, 1.16% DMSO, v/v), segregated into 4 cohorts [12], [13]. Acutely-treated mice received their first injection 45 minutes prior to ischemia(day 0) followed by daily injections on days 1–2 post-ischemia, while mice in the delayed cohort were dosed on post-stroke days 3–6. A combined-treatment group was dosed daily for a total of 7 days beginning 45 minutes prior to ischemia (day 0). Vehicle-treated control animals received 7 daily injections of PBS on the same regimen as the combined-treatment cohort. All treatment cohorts received a balance of vehicle injections to control for injection volume. All mice also received daily intraperitoneal BrdU (50 mg/kg). All injections were performed in a blinded fashion.

**Figure 3:**
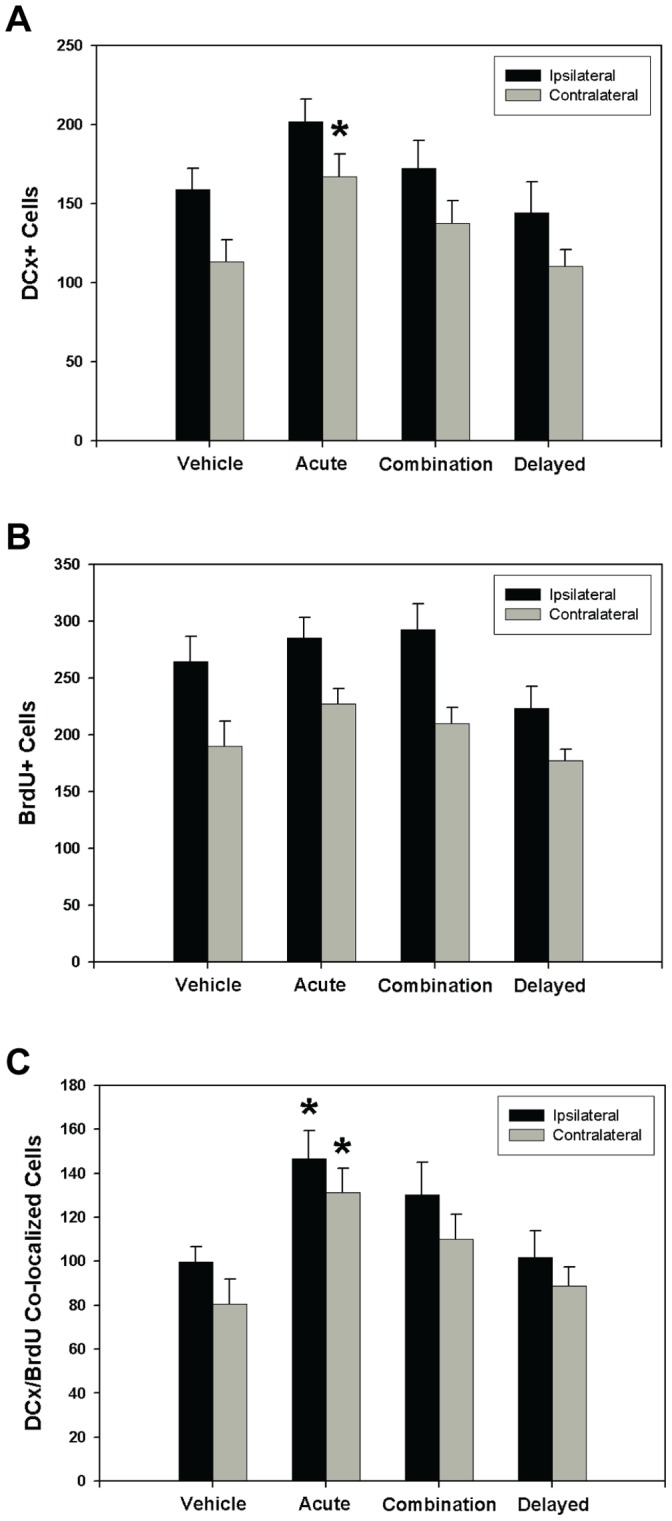
Acute C3aRA administration promotes post-ischemic neurogenesis in the SVZ. The effect of C3aR antagonism on the extent of ischemia-induced neurogenesis in the SVZ at 7 days post-reperfusion was assessed by immunostaining for both DCx and BrdU. Analysis of stereological cell-counts in the ipsilateral and contralateral SVZ revealed significant differences between treatment groups. Animals in the acute treatment cohort demonstrated significant increases (p<0.05) of DCx+ cells in the contralateral SVZ relative to both vehicle and delayed treatment cohorts (vehicle: n = 12, acute: n = 18, combined: n = 14, delayed: n = 14) (**A**). No significant differences in the total BrdU+ count were observed either in the ipsilateral or contralateral SVZ (vehicle: n = 12, acute: n = 18, combined: n = 14, delayed: n = 14) (**B**). DCx/BrdU co-localized cells were significantly increased (p<0.05) in the acute treatment cohort relative to both vehicle and delayed cohorts (vehicle: n = 12, acute: n = 18, combined: n = 14, delayed: n = 14). (**C**). This comparison was significant in both ipsilateral and contralateral SVZ.

### Murine Stroke Model

Previous work investigating the functional benefit of C3a receptor antagonism evaluated outcome at 24 hours [7], [14]. In the present experiment, we employed a variation in our model of transient MCAO that allowed for an extended 7-day survival by reducing the occlusion period to 30 minutes. This modification provides consistent infarcts that primarily involve subcortical regions. Despite a mild increase in total infarct variability resulting primarily from increased cortical infarct variance, this modification allows for extended survival in conjunction with a reliable neurologic deficit that may be assessed as a functional endpoint in long-term studies, and is critical in evaluating therapeutic strategies for stroke.

**Figure 4:**
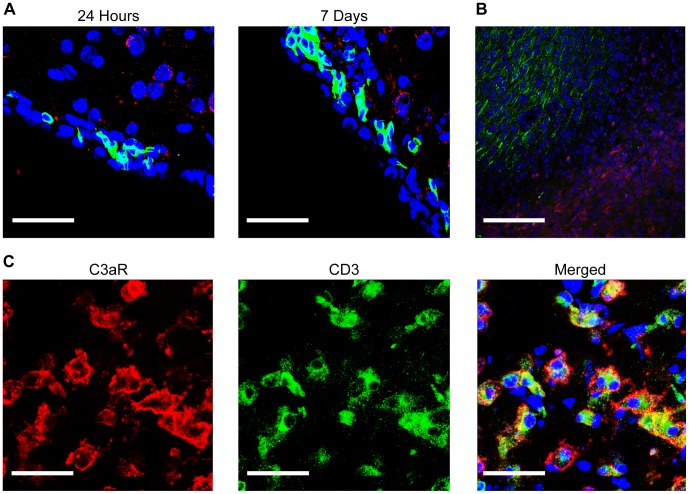
C3aR is robustly expressed on the surface of CD3+ cells in the ischemic region. C3aR is not expressed on cells in the SVZ at 24 hours post-MCAO. Immunostaining of the SVZ at 24 hours and 7 days post-MCAO reveals several migrating neuroblasts (DCx+, green) without any significant C3aR immunopositivity (red) (**A**). Images are representative of findings from at least 4 animals per experiment. Scale bars = 75 µm. Examination of the infarct border at 7 days at low-power reveals a massive influx of cells bearing C3a receptor (red) in the region of the infarct, counterstained with Nissl (blue), depicted by a loss of MAP2 staining (green) (**B**). Scale bars = 170 µm. Higher-power images in the region of the infarct more clearly demonstrate cells bearing C3aR (red), many of which demonstrate robust expression of CD3 (green) (**C**). Merged channels depict unequivocal co-localization of C3aR with CD3 (yellow) in the majority of cells observed. Scale bars = 75 µm.

Mice were subjected to transient middle cerebral artery occlusion (MCAO) as described previously [7], [15] using 1.5% isoflurane in a 70% N_2_O/30% O_2_ gas mixture, and a heat-blunted, silicon-coated 7–0 nylon monofilament. Reperfusion was achieved by withdrawing the filament following 30 minutes of ischemia. Animals were only included if occlusion suppressed blood flow <30% of baseline, and filament withdrawal resulted in prompt restoration of flow. Animals were sacrificed at either 24 hours or 7 days, and brains were harvested and prepared for histology as described. Cerebral blood flow measurements were recorded and intra-operative temperatures were controlled as described in Methods S1.

**Figure 5:**
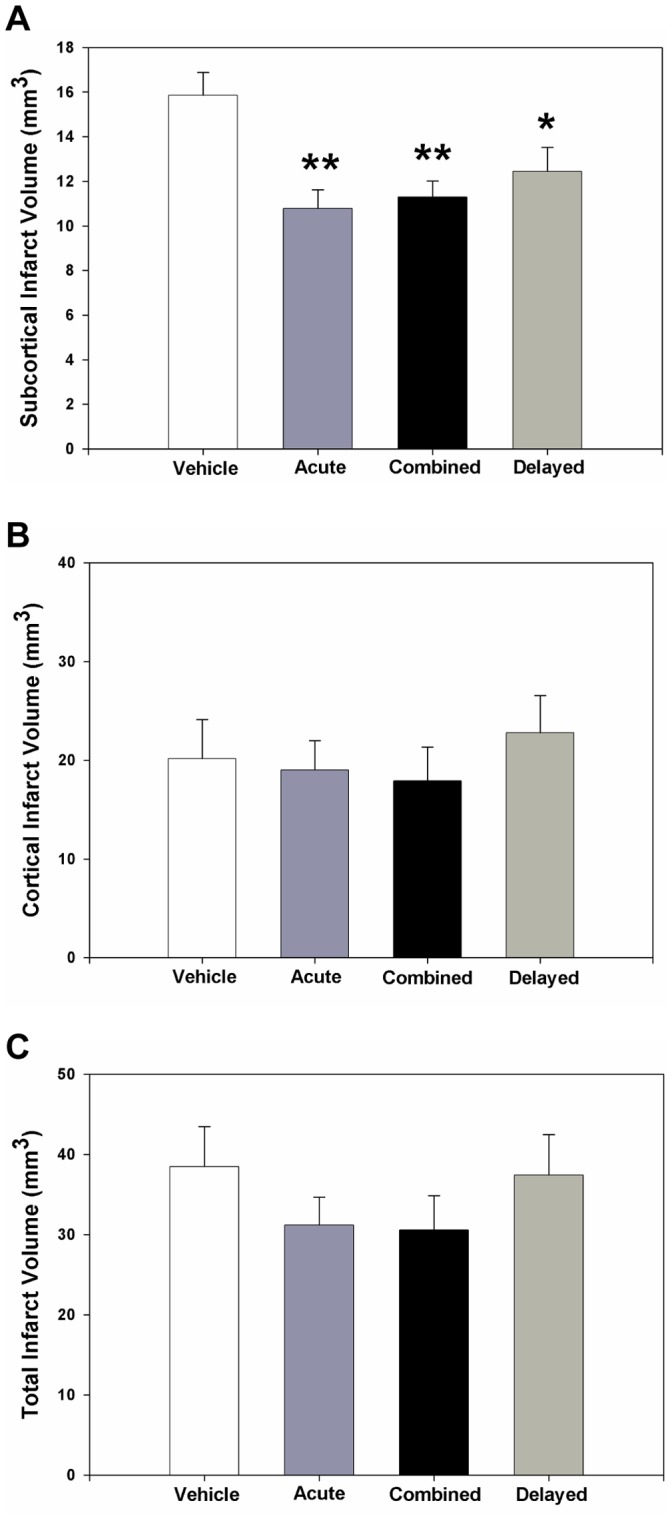
Antagonism of the C3aR provides sustained histologic neuroprotection. Infarct volume was determined by integrating serial coronal H&E stained sections. Significantly decreased volume of subcortical infarction was observed with C3aRA treatment relative to vehicle in all cohorts (vehicle: n = 21; acute: n = 24, combined: n = 23, delayed: n = 23) (p<0.01**, p<0.05*) (**A**). There were no significant reductions in cortical (**B**) and total infarct volume (**C**) observed.

### Evaluation of Basal Neurogenesis

Mice received intraperitoneal injections of C3aRA twice daily for 10 days. The high-dose cohort received 20 mg/kg per injection, for a total of 40 mg/kg/day [11]. The low-dose cohort received 1 mg/kg in a single dose, with an additional vehicle injection to control for volume. Control mice received twice-daily injections of vehicle. All mice also received intraperitoneal BrdU (200 mg/kg) for seven days. Injections were performed by a blinded investigator. On day 10, brains were harvested for immunohistochemistry as described below.

**Table 1:** Mortality Rates In Each Treatment Cohort.

Cohort	Deaths	n	Mortality (%)
Vehicle	11	32	34.4
Acute	3	27	11.1
Combined*	1	24	4.2
Delayed	6	29	20.7

Overall χ2 test value for 2×4 contingency table = 9.6; p = 0.02.

* p = 0.008 relative to vehicle.

### Histology

Following transcardiac PBS perfusion, brains were harvested, fixed in 4% paraformaldehyde, and cryoprotected. Brains were frozen in optimal cutting temperature compound (Sakura Finetek US, Torrance, CA, USA) at −80°C, and cut into coronal sections (20 *µ*m). Sections were blocked with secondary antibody-appropriate serum with PBS containing 0.2% Triton X-100 for 30 minutes at room temperature. Sections were incubated in primary antibodies diluted in PBS containing 0.2% Triton X-100 overnight at 4°C. Sections were then washed and incubated with secondary antibodies for 1 h. Nissl was used for counterstaining. Sections were then mounted on slides in Vectashield (Vector Laboratories, Burlingame, CA, USA) and visualized using a Bio-Rad 2000 confocal laser-scanning device (Bio-Rad, Hercules, CA, USA) with a Nikon E800 microscope (Nikon, San Diego, CA, USA). A list of primary antibodies can be found in Methods S2.

**Figure 6:**
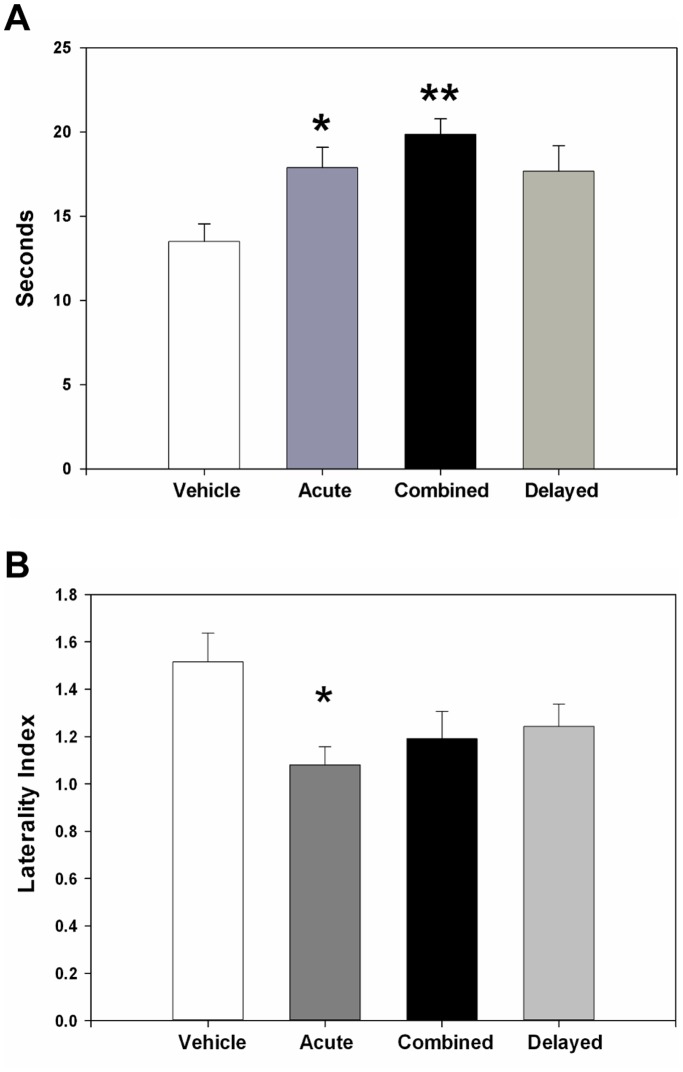
C3aR antagonism improves neurological function following reperfused stroke. The Morris water maze was used to evaluate navigational memory at 7 days post-stroke. Significant improvement (vehicle: n = 21, acute: n = 24, combined: n = 23, delayed: n = 23) was observed in both acutely and combined-treated mice (p = 0.04* and p = 0.002** respectively) (**A**). The corner turn test was employed to evaluate post-ischemic sensorimotor function and demonstrated significantly (p = 0.02) improved outcome in the acutely-treated animals (vehicle: n = 25, acute: n = 26, combined: n = 24, delayed: n = 28) (**B**).

### Confocal Stereology and Quantification

The optical fractionator technique was employed for cell-count quantification [16]. Images were captured with a confocal microscope at the resolution 1024×1024 pixels under a x40 objective, with a counting frame of 295 µm×295 µm. Z-sectioned merged images were obtained from a stack of adjacent 10 sections (step 1 µm). The number of DCx and BrdU immunopositive cells were quantified in serial coronal sections obtained at 500 µm intervals from every studied brain in the granular cell layer of the olfactory bulb (OB)(3 sections per brain, bregma levels +4.5 to +3.5 mm), in the SVZ (4 sections per brain, bregma levels +1.5 to 0 mm) and the subgranular zone (SGZ) of the dentate gyrus (5 sections per brain, bregma levels -1.0 to −3.0 mm). For phenotypic analyses of BrdU-positive cells, we used double immunostaining for BrdU and either DCX, NeuN or GFAP. At least 100 BrdU-positive cells were analyzed for each marker per animal. The number of BrdU-, DCX-, C3aR- and CD3-positive cells in the peri-infarct area of the frontal cortex and striatum (bregma levels +2 mm to −0.5 mm) were quantified on the images taken with the same microscope settings as above. At least four images from each area of interest in each of six sections (500 µm apart) were used.

Cell counting was performed by a blinded observer. Results for ischemia-induced neurogenesis (SVZ, OB, cortex, striatum), basal neurogenesis (SVZ, OB, SGZ) and CD3/C3aR+ in the peri-infarct region were presented as total cell-counts.

### Determination of Infarct Volume

Serial coronal sections at 0.5 mm coronal intervals were stained with H&E. Areas of infarct were delineated at high-power using a Nikon Labophot with Nikon DS-5M digital sight camera system and quantified using a standard computer-assisted image analysis technique (Image Pro Plus 4.5, Media Cybernetics, Bethesda, MD). Subcortical regions were discriminated from the overlying cortex on each coronal section by a line approximating the corpus callosum, cingulum, and external capsule.

### Neurofunctional Assessment

Navigational memory and learning were evaluated using the Morris-water-maze, as previously described [17], [18] (Methods S3). Sensorimotor impairment was analyzed using the corner-turn test, as previously described [18] (Methods S4). The laterality index (LI) and normalized LI were then calculated as previously published [19], according to the formula: LI = (number of right turns-number of left turns)/(total number of turns). The LI for the day before surgery (LI_BS_) and each of the post-surgery days was calculated and normalized using the formula: Normalized LI = (LI+2)/(LI_BS_+2). Neurofunctional assessments were performed by a blinded investigator.

### Statistical Analysis

Data are presented as mean±standard error of the mean (SEM). Between-group differences were evaluated using one-way analysis of variance (ANOVA) with post-hoc Dunnett or Tukey tests for continuous variables; and χ^2^ and Fisher’s exact test for categorical variables. Kruskal-Wallis with post-hoc Dunn’s Test was utilized for non-parametric data. Values were considered significant when P≤0.05. Analyses were carried out using commercially-available software (SPSS Statistics 17.0, SPSS Inc., Chicago, IL).

## Results

### Experimental Cohorts

Animals in each cohort were balanced in terms of age and size (vehicle: 24.8±0.1 g, acute: 25.1±0.2 g, combined: 25.0±0.2 g, delayed: 25.0±0.2 g). There were no significant differences in CBF between the cohorts at any time-point evaluated.

### Antagonism of the C3a-receptor Exhibits Dose-dependent Effects on Basal Neurogenesis in the SVZ

To demonstrate a dose-dependent effect of C3aRA administration on basal neurogenesis, the present study compared three different doses (40 mg/kg/day, 1 mg/kg/day, and vehicle), and evaluated animals following 10 days of administration. Immunostaining of the SVZ was performed for DCx, a phenotypic marker of immature neurons, and BrdU, a marker of cell proliferation (Figure 1A, B; Figure 2). Animals receiving 40 mg/kg of C3aRA daily demonstrated a suppression of BrdU^+^ cells relative to the 1 mg/kg cohort (p = 0.01) (Figure 1C). BrdU/DCx++ (double-positive) cells in the SVZ were also suppressed in the high-dose cohort (Vehicle: 107.9±9.18, 1 mg/kg: 124.5±7.72, 20 mg/kg: 96.4±7.22; p = 0.05) (Figure 1D). While a similar relationship in cell-count was also observed in the OB, these values did not achieve statistical significance (data not shown).

### A Neuroprotective Dose of C3aRA Supports the Proliferation of Migrating Neuroblasts in the SVZ Following Reperfused Stroke

Given the proposed role for C3a in stimulating post-ischemic neurogenesis [11], we also evaluated baseline expression and the effect of low-dose C3aR antagonism (Figure S1 A-D) on ischemia-induced neurogenesis in the SVZ at 7 days post-reperfusion. Stereological techniques were employed to analyze counts of DCx+ and BrdU+ cells in the SVZ of both the ipsilateral and contralateral hemispheres (Figure 3 A-C). Analyses of DCx/BrdU co-labeled cells in the ipsilateral SVZ revealed that acutely treated animals (146.7±12.6) demonstrate significant increases in double-labeled cells relative to both vehicle (99.8±7.0) and delayed-treatment (101.8±12.2) cohorts (p<0.05 for both comparisons). In the contralateral SVZ, whereas no significant differences were observed between cohorts in the number of BrdU labeled cells, acutely-treated animals (166.9±14.4) demonstrated a significant increase in DCx-labeled cells relative to both vehicle (113.1±13.9; p = 0.02) and delayed-treatment (110.3±10.7; p = 0.04) cohorts. Furthermore, DCx/BrdU co-labeled cells were observed more frequently in the acute cohort (131.4±11.0) relative to both vehicle (80.7±11.2; p = 0.009) and delayed-treatment (88.6±8.8; p = 0.02) cohorts. There were no significant differences between the combination treatment group and the remainder of the cohorts for all analyses.

Taken together, these data suggest that acutely administered low-dose C3aR blockade promotes ischemia-induced neuronal precursor cell proliferation in the SVZ at 7 days post-ischemia.

### C3aR is Expressed on Granulocytes in the Ischemic Region at 24 hours and on Infiltrating T-lymphocytes at 7 Days following Reperfused Stroke

To investigate the cellular localization of C3aR in the setting of reperfused stroke in our model, immunohistochemistry was performed on brain tissue samples obtained at both 24 hours as well as 7 days post-reperfusion. At 24 hours, staining for C3aR was evident in the ischemic region on the surface of cells that strongly co-expressed Ly6G, a granulocyte-specific marker (Figure S2). This observation, previously reported by our group [7], was confirmed using multiple commercially available anti-C3aR antibodies. Importantly, no cells bearing the C3aR were observed outside of the ischemic region at 24 hours, including in the SVZ, SGZ, OB or in the contralateral non-ischemic hemisphere (Figure 4A). Even at 7 days post-ischemia, the lack of immunopositivity in regions known to produce precursor cells was maintained (Figure 4A). Instead, a large number of cells positive for C3aR were again observed in the region of the infarct (Figure 4B). This population, which did not express Ly6G, demonstrated instead robust expression of CD3, a cell-surface marker for T-lymphocytes (Figure 4C). In fact, the vast majority of C3aR+ cells observed at this time-point strongly expressed CD3. Thus, a robust infiltration of C3aR− expressing lymphocytes occurs by 7 days post-ischemia, representing a marked shift in the population of inflammatory cells expressing the C3aR during the subacute phase of stroke.

### C3a Receptor Antagonism Suppresses the Infiltration of T-lymphocytes into the Ischemic Region

The role of C3a receptor antagonism on the modulation of the CD3+ cellular infiltrate at this subacute time-point was then evaluated in vehicle, acute, and combined-treatment cohorts. Numerous cells expressing C3aR and CD3 were observed in the ischemic region (Figure 4B-C). The combined C3aRA regimen significantly suppressed the number of cells expressing both C3aR (vehicle: 28.3±3.0; acute: 19.4±1.8; combined: 18.6±3.2; p<0.05 for vehicle vs. combined) (Figure S3A) and CD3 (vehicle: 35.5±3.3; acute: 26.1±3.5; combined: 21.4±3.5; p<0.05 for vehicle vs. combined) (Figure S3B) in the ischemic territory at 7 days. By comparison, no significant suppression of cells expressing C3aR or CD3 was observed in mice treated only during the acute period (Figure S3A-B). Therefore, effective suppression of CD3+ cells requires ongoing administration of C3aRA beyond the initial 72 hours post-reperfusion.

### C3a Receptor Antagonism Ameliorates Subcortical Infarction, Reduces Mortality and Improves Functional Outcome Following Reperfused Stroke

Analysis of serial H&E-stained coronal sections (Figure S4A) at 7 days revealed significantly decreased volume of subcortical infarction (mm^3^) following C3aRA administration in all treatment groups compared to vehicle (vehicle: 15.8±1.0; acute: 10.8±0.8, p<0.01; combined: 11.3±0.7, p<0.01; delayed: 12.4±1.1, p<0.05) (Figure 5A). These findings indicate that C3aRA administration decreases, rather than simply delays, tissue injury, and that this protection occurs predominantly in subcortical regions in this model of MCAO.

Mortality was also significantly reduced with C3aRA treatment (Table 1, Figure S4B, χ^2^ = 9.6, p = 0.02). Subsequent post-hoc analysis revealed a trend towards improved mortality in acutely-treated mice (11.1% vs. 34.4%; p = 0.06) and a greater than 8-fold reduction in mortality in the combination treatment cohort (4.2% vs. 34.4%; p = 0.008).

We also evaluated the effect of C3aRA administration on neurofunctional outcome in our MCAO model. The Morris Water Maze evaluates spatial navigation, learning, and memory: complex functions that reside in the hippocampus, striatum, and cortex [17], [20]. Using this test, we demonstrated significant improvement in both the acute (p = 0.04) and combined (p = 0.002) cohorts relative to vehicle (vehicle: 13.5±1.3; acute: 17.9±1.2, p = 0.04; combined: 19.8±1.2, p = 0.002; delayed: 17.6±1.2) (Figure 6A). Post-stroke sensorimotor dysfunction was also evaluated using the corner-turn test [21]. This revealed significantly improved sensorimotor function in acutely-treated animals (vehicle: 1.5±0.1; acute: 1.1±0.08; combined: 1.2±0.1; delayed: 1.2±0.1; p = 0.02) (Figure 6B).

## Discussion

Our group has previously employed gene-deleted mice in combination with specific pharmacologic inhibitors to identify C3 as the central mediator of complement-induced acute cerebral ischemic injury following reperfused stroke in mice [14]. As with most prior studies investigating the pathogenesis of post-ischemic cerebral inflammation, this work limited evaluation of outcome to 24 hours [7], [14]. In the present study, we investigated the effects of targeted C3a receptor antagonism on post-ischemic neurogenesis, histologic infarction, lymphocyte infiltration, and behavioral recovery in a stroke model which permits extended post-ischemic survival.

The recent description of complement as a regulator of neurogenesis has enormous implications for the potential translation of anti-complement strategies [11]. A close examination of the existing literature linking complement to basal and post-ischemic neurogenesis reveals various roles for complement activation in these processes. First, Rahpeymai *et al.* published a study reporting that neural progenitor cells and immature neurons express both C3aR and C5aR [11]. These authors also observed a reduction in proliferating neural progenitors and migrating neuroblasts in the SGZ and OB of animals treated with C3aRA as well as in mice genetically-deficient for C3aR [11]. Furthermore, C3-null mice subjected to middle cerebral artery occlusion (MCAO) demonstrate suppressed ischemia-induced neurogenesis and larger infarcts when assessed at delayed time-points. This study suggests that chronic inhibition of the C3a/C3aR axis would suppress neurogenesis and thus potentially negatively impact functional outcome.

A subsequent study by the same group [22], however, does not support such a clear role for C3a in neurogenesis. This second study utilized transgenic mice that over-express C3a in the central nervous system under the control of the GFAP promoter. Interestingly, these C3a/GFAP transgenic mice did not demonstrate increased basal neurogenesis in either the SVZ or the SGZ relative to wild-type controls. The authors conclude that the C3a peptide produced by these mice is likely not generated in sufficient quantities in the neurogenic niche under basal conditions; however, they do not provide evidence to support this contention. A more recent study by Morimaya *et al.* suggests an inhibitory role for complement in basal hippocampal neurogenesis [23]. In this study, the authors find that CR2 genes are expressed in neural progenitor cells, and that administration of CR2 ligands, including C3d, reduces the proliferation of wild-type primary mouse neural progenitor cells *in vitro*. They follow with *in vivo* experiments utilizing direct injection of C3d into the dentate gyrus of wild-type mice to demonstrate a decrease in proliferating neuroblasts relative to saline-injected animals. Based on the available literature, the influence of complement on neurogenesis is complex, and demands careful analysis of each complement component across varying time-points in a specific a disease model.

In the present study, low-dose C3aRA administration limited to the acute post-stroke period increases the proliferation of migrating neuroblasts in the SVZ. The differences observed between our study and that of Rahpeymai *et al.* are likely explained in part by variations in experimental protocols [11]. First, Rahpeymai *et al.* employ models of permanent stroke, which are known to induce a substantially different pathological response than that of reperfused stroke [24]. Furthermore, the dose of C3aRA utilized by Rahpeymai *et al.* in their work was forty times greater than the low-dose utilized in our study and in previous studies demonstrating a neuroprotective benefit in stroke [7], [14]. In fact, we demonstrate that a high-dose regimen of C3aRA suppresses both BrdU+ and DCx/BrdU++ cells in the SVZ relative to the low-dose regimen, suggesting a dose-dependent effect of C3aRA administration on neural progenitor proliferation. This dose-dependence suggests that complement inhibition may be pharmacologically targeted to promote post-ischemic progenitor cell proliferation. It is important to note that we did not observe a corresponding increase in newly-formed mature neurons either in the SVZ or in the peri-infarct region. This likely relates to our use of a 7-day sacrifice time-point, as the process of neuroblast migration from the SVZ to the ischemic territory and subsequent maturation is not complete by this time [25]. Additionally, we did not observe a significant difference between the number of DCx/BrdU++ cells in the ischemic and non-ischemic hemispheres in any of the treatment cohorts. Given that the absolute value of the DCx/BrdU++ cell count in the ipsilateral hemisphere of each cohort is larger than that of the contralateral hemisphere, the lack of significance between these comparisons might simply reflect a lack of statistical power. Alternatively, while an increase in DCX+BrdU+ cells in the hemisphere ipsilateral to an ischemic insult has been described, increased neurogenesis contralateral to an ischemic lesion has also been previously reported [26], [27]. This increase in contralateral neurogenesis may mask an anticipated difference between hemispheres, and may go unnoticed in studies that only examine cell counts in the ipsilateral hemisphere. Finally, the proliferation of cells in the ischemic SVZ has been reported as maximal later than 7 days post-ischemia, and thus a significant inter-hemispheric difference may ultimately be noted at a later time-point [28]. Future experiments are planned examining the functional and histologic effects of C3aRA administration at later time-points.

The use of an *in vivo* stroke model also complicates the analysis of the effects of complement inhibition on neurogenesis. It is well-established that post-ischemic neurogenesis is subject to a complex interplay between complement activation, cytokine release, and other inflammatory processes [29]. In addition to modulating neurogenesis directly, complement activation may also serve to inhibit neurogenesis indirectly through upregulation of inflammatory cytokines. For example, recent work demonstrates the detrimental effect of IFN-γ and TNF-α on rat neuronal progenitor cell survival and proliferation [30]. Thus, the direct effect of C3a/C3aR blockade on neurogenesis cannot be dissociated from its powerful anti-inflammatory effect in a stroke model. As it has been established that inflammation is a more prominent characteristic of reperfused stroke than non-reperfused stroke, the known suppressive effect of inflammatory cytokines on neurogenesis may figure prominently in our model [24].

The histologic findings of our study also support an indirect influence of C3a on neural progenitors, and serve to identify a novel mechanism for complement-mediated ischemic injury beyond the acute phase of stroke. Using confocal microscopy, C3aR antigen was observed *only* on infiltrating inflammatory cells in the ischemic territory. This calls into question a direct effect of C3aR on SVZ neurogenesis. At 24 hours, C3aR expression was restricted to the surface of granulocytes. By 7 days, however, a distinct population of cells bearing the CD3 marker was observed, the majority of which expressed the C3a-receptor. Daily administration of C3aRA suppressed the infiltration of both CD3+ and C3aR+ cells. To our knowledge, this represents the first report of the C3aR localized to infiltrating T-lymphocytes in the brain, and suggests that C3a plays a role in modulating post-ischemic T-cell infiltration. As T-cells infiltrate the ischemic region in a delayed fashion relative to granulocytes [31], this finding raises the possibility of an extended therapeutic window for anti-complement neuroprotective strategies. In fact, when C3aRA administration was delayed until 72 hours post-ischemia and continued to the sacrifice time-point of 7 days, significant reductions in subcortical injury were also observed. Furthermore, we observed more robust functional neuroprotection and reduction in mortality when acutely administered, low-dose C3aRA administration was continued through the subacute phase (combined cohort). This indicates that deleterious complement-mediated cerebral inflammation persists into the subacute phase, and that targeting these deleterious subacute processes may afford additional neuroprotection. Additionally, it has been reported that activated T-cells may suppress neural progenitor cell proliferation and differentiation [32]. Therefore, a suppressive effect of C3aRA on T-cell infiltration may also indirectly promote neurogenesis through as yet undefined mechanisms.

We must acknowledge several limitations of our study. First, conclusions derived from this work cannot be extrapolated to models of permanent ischemia. We believe that reperfused stroke represents a clinically-relevant experimental model, particularly given the recent extension of the therapeutic window for intravenous thrombolysis, as well as the rapid development of endovascular techniques of reperfusion [33]. Furthermore, as these experiments were primarily designed to elucidate the mechanisms C3a/C3aR-mediated injury, we did not perform post-ischemic dosing regimens, which are ultimately critical for translation of anti-complement strategies. Additionally, as neurofunctional recovery and neurogenesis continue into the chronic phase of stroke, further work is necessary to definitively establish the role for C3a/C3aR in neurorecovery at later post-ischemic time-points.

In conclusion, despite reports of a positive regulatory role for complement in neurogenesis, targeted complement inhibition through low-dose antagonism of the C3a receptor actually promotes the proliferation of migrating neuroblasts in the SVZ following reperfused stroke. Furthermore, C3aR is expressed by infiltrating T-lymphocytes in the ischemic region at 7 days post-ischemia, and C3aRA administration attenuates the infiltration of these cells. This uncovers a novel role for complement in the modulation of the lymphocyte infiltrate in the subacute phase of stroke evolution. Additionally, the functional and histologic neuroprotection associated with C3aRA administration is sustained when evaluated at an extended time-point. Although further work is necessary to characterize the mechanisms of complement-mediated inflammatory signaling and its effect on post-ischemic neurogenesis, as well as the relationship between neurogenesis and functional outcome, targeted pharmacologic inhibition of complement may ultimately represent an effective strategy for the treatment of stroke.

## Supporting Information

Figure S1 **Acute C3aRA treatment stimulates proliferation of migrating neuroblasts in the SVZ at 7 days post-ischemia.** Ischemia-induced neurogenesis is depicted by representative immunostaining of the SVZ of animals treated with vehicle (**A**), acute (**B**), combined (**C**), and delayed (**D**) dosing regimens of C3aRA. BrdU is represented in the green channel, DCx in the red channel, and co-localized cells appear yellow. Images are representative of findings from at least 4 animals per experiment. Scale bar = 200 µm.
(TIF)

Figure S2 **C3aR is expressed on the surface of infiltrating granulocytes in the ischemic region.** Immunostaining of tissue sections obtained from animals sacrificed at 24 hours reveals numerous cells present in the area of the developing infarct that express C3aR (red) as well as the granulocyte-specific marker Ly6G (green). Merged channels demonstrate co-localization of C3aR with Ly6G (yellow). The tissue was counter-stained with Nissl (blue).
(TIF)

Figure S3 **Sustained C3aRA treatment suppresses CD3 infiltration into the ischemic area post-reperfusion.** Quantitative analysis (representative of staining from n = 7 animals per cohort) reveals a significant decrease in the number of C3aR+ (p<0.05) cells (**A**) as well as CD3+ (p<0.05) cells (**B**) in the combined treatment cohort relative to vehicle.
(TIF)

Figure S4 **Antagonism of the C3aR reduced mortality following reperfused stroke.** Infarct volume was determined by integrating serial coronal H&E stained sections. Representative coronal section obtained from a vehicle-treated animal depicting extensive infarction incorporating both subcortical and cortical regions (**A**). On this coronal section, subcortical and cortical regions are separated by a line approximating the corpus callosum, cingulum and external capsule. Mortality rates over 7 days were significantly improved following C3aRA administration, with post-hoc analysis demonstrating a trend towards improved mortality in acutely-treated mice (p = 0.06), and a significant reduction in mortality in the combined treatment cohort (p = 0.008*) (**B**).
(TIF)

Methods S1 **Cerebral Blood Flow Measurements and Temperature Control.**
(DOCX)

Methods S2 **Immunohistochemistry Antibodies.**
(DOCX)

Methods S3 **Morris Water Maze Procedure.**
(DOCX)

Methods S4 **Corner-Turn Test Procedure.**
(DOCX)
