# Supplementary figures and images for: Complement Inhibition Promotes Endogenous Neurogenesis and Sustained Anti-Inflammatory Neuroprotection following Reperfused Stroke
Source: PLoS One. 2012 Jun 26;7(6):e38664. doi: 10.1371/journal.pone.0038664 (PMC3383680; doi:10.1371/journal.pone.0038664)

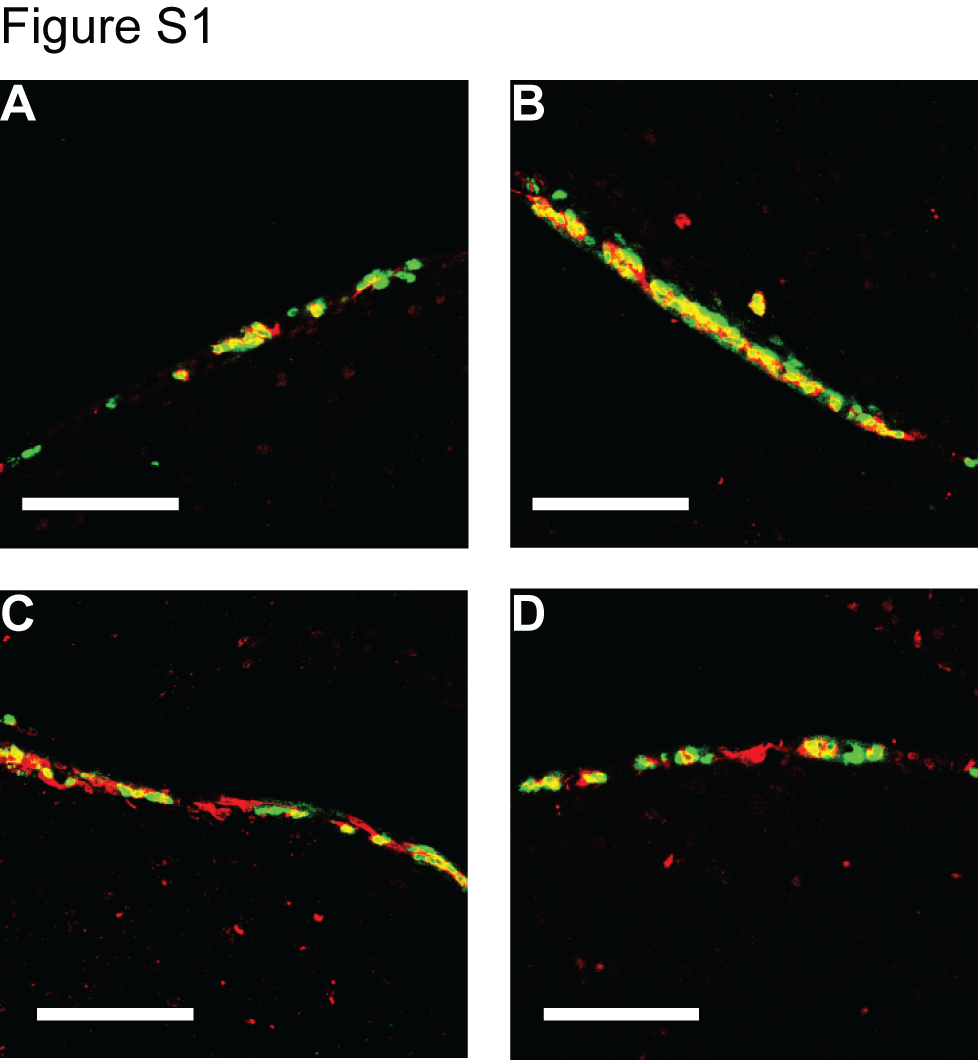

Supplement: Figure S1 — Acute C3aRA treatment stimulates proliferation of migrating neuroblasts in the SVZ at 7 days post-ischemia. Ischemia-induced neurogenesis is depicted by representative immunostaining of the SVZ of animals treated with vehicle (A), acute (B), combined (C), and delayed (D) dosing regimens of C3aRA. BrdU is represented in the green channel, DCx in the red channel, and co-localized cells appear yellow. Images are representative of findings from at least 4 animals per experiment. Scale bar = 200 µm. (TIF) [file pone.0038664.s001.tif]

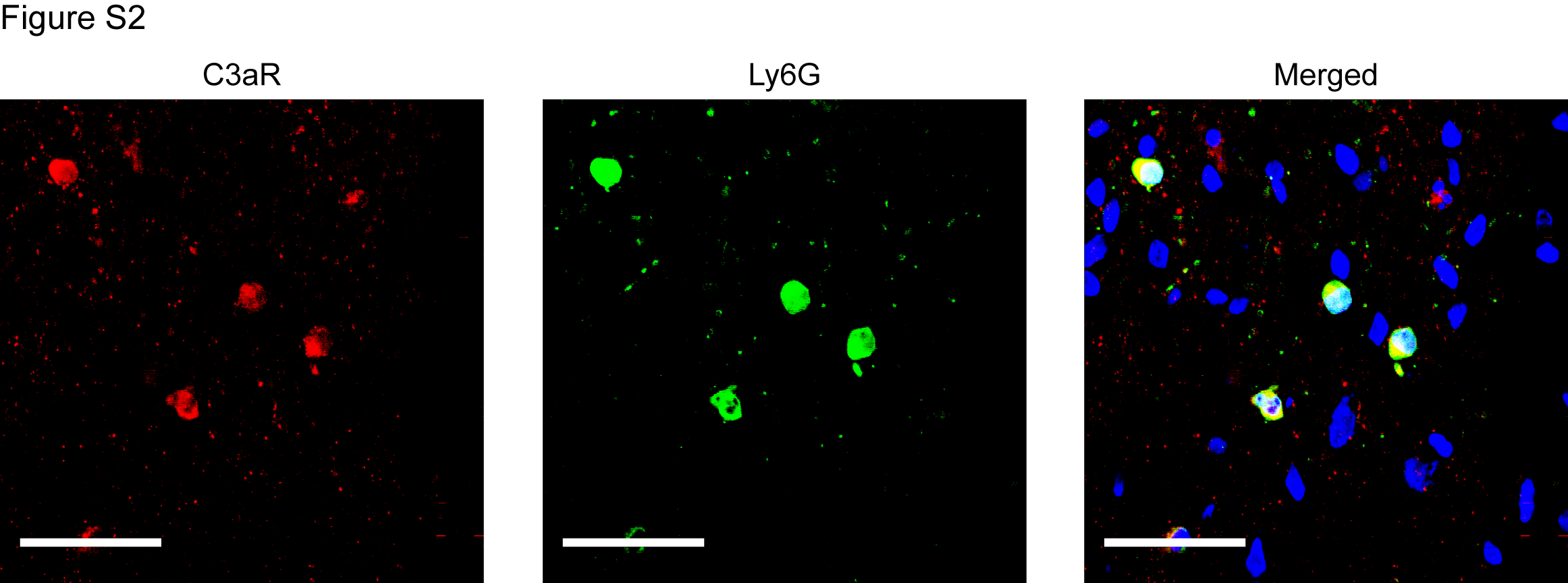

Supplement: Figure S2 — C3aR is expressed on the surface of infiltrating granulocytes in the ischemic region. Immunostaining of tissue sections obtained from animals sacrificed at 24 hours reveals numerous cells present in the area of the developing infarct that express C3aR (red) as well as the granulocyte-specific marker Ly6G (green). Merged channels demonstrate co-localization of C3aR with Ly6G (yellow). The tissue was counter-stained with Nissl (blue). (TIF) [file pone.0038664.s002.tif]

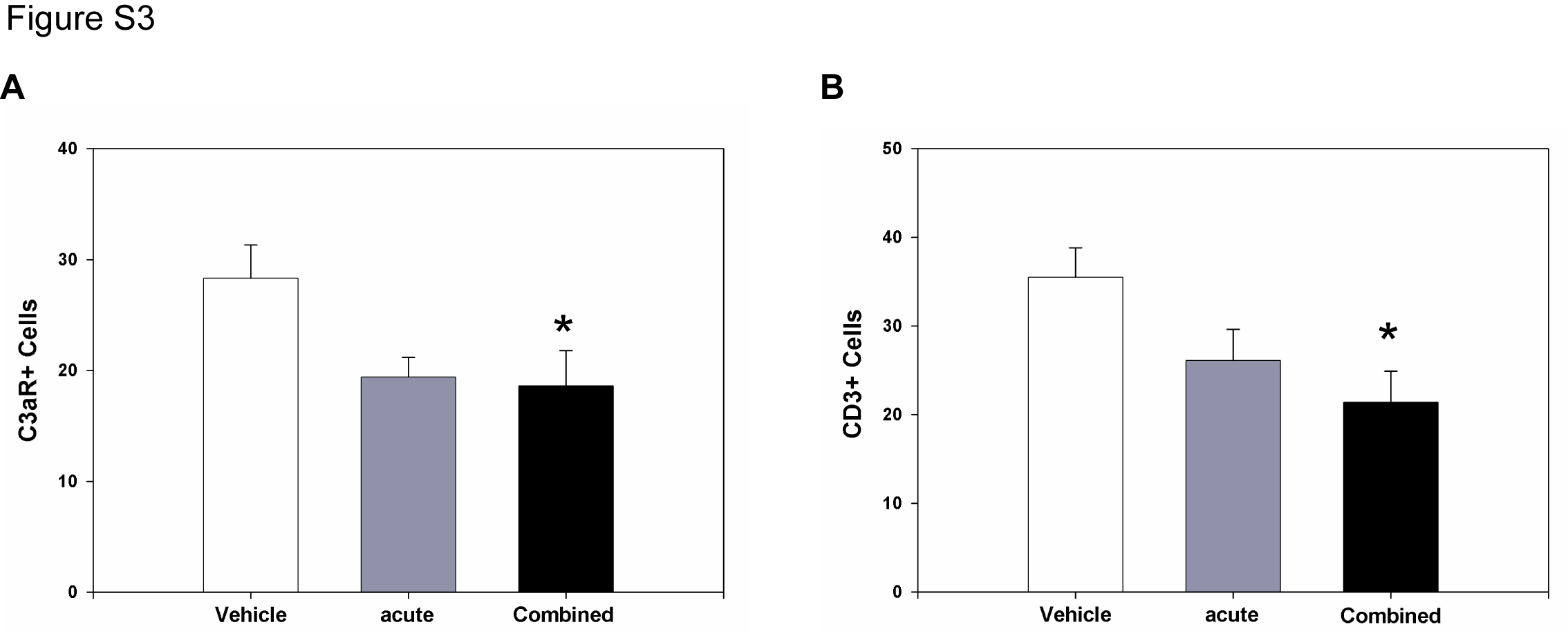

Supplement: Figure S3 — Sustained C3aRA treatment suppresses CD3 infiltration into the ischemic area post-reperfusion. Quantitative analysis (representative of staining from n = 7 animals per cohort) reveals a significant decrease in the number of C3aR+ (p<0.05) cells (A) as well as CD3+ (p<0.05) cells (B) in the combined treatment cohort relative to vehicle. (TIF) [file pone.0038664.s003.tif]

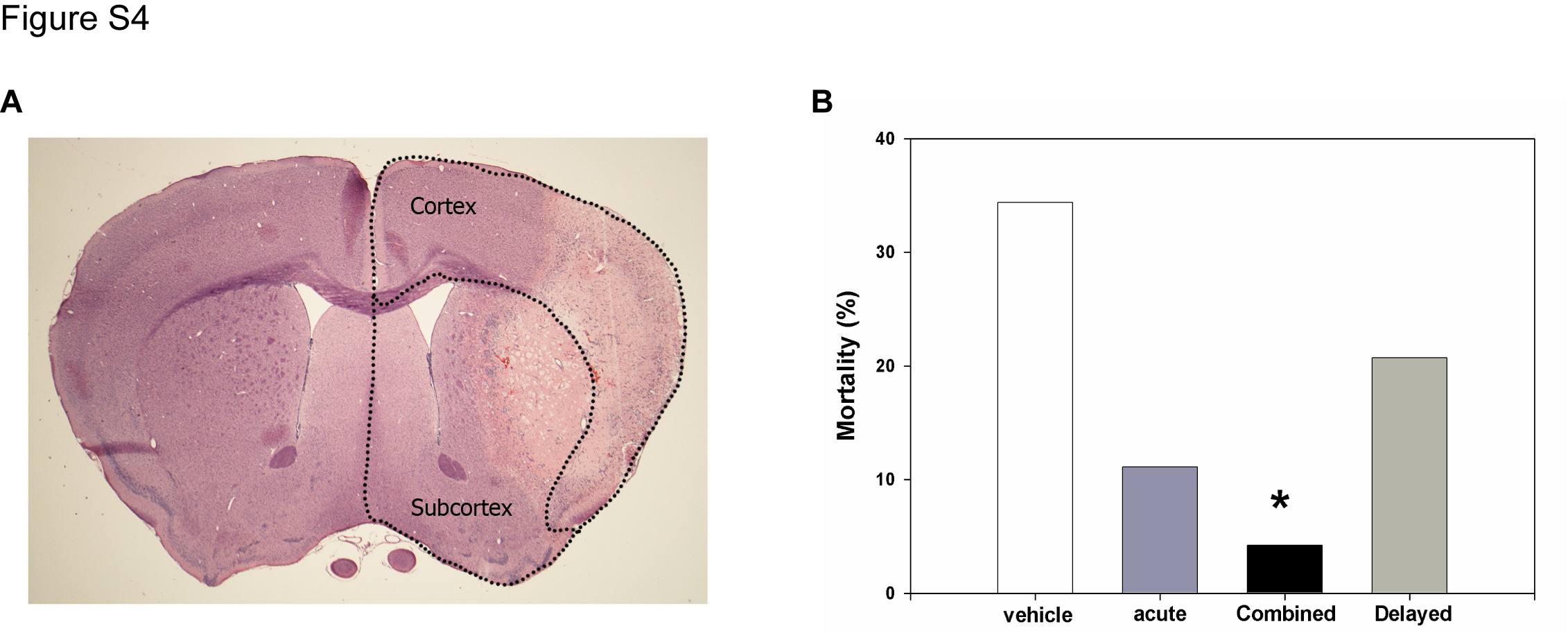

Supplement: Figure S4 — Antagonism of the C3aR reduced mortality following reperfused stroke. Infarct volume was determined by integrating serial coronal H&E stained sections. Representative coronal section obtained from a vehicle-treated animal depicting extensive infarction incorporating both subcortical and cortical regions (A). On this coronal section, subcortical and cortical regions are separated by a line approximating the corpus callosum, cingulum and external capsule. Mortality rates over 7 days were significantly improved following C3aRA administration, with post-hoc analysis demonstrating a trend towards improved mortality in acutely-treated mice (p = 0.06), and a significant reduction in mortality in the combined treatment cohort (p = 0.008*) (B). (TIF) [file pone.0038664.s004.tif]
