# Supplementary material for: Complement Inhibition Promotes Endogenous Neurogenesis and Sustained Anti-Inflammatory Neuroprotection following Reperfused Stroke
Source: PLoS One. 2012 Jun 26;7(6):e38664. doi: 10.1371/journal.pone.0038664 (PMC3383680; doi:10.1371/journal.pone.0038664)
Supplement: Methods S1 — Cerebral Blood Flow Measurements and Temperature Control. (DOCX) [file pone.0038664.s005.docx]

**Methods S1. Cerebral Blood Flow Measurements and Temperature Control.** Continuous transcranial cerebral blood flow (CBF) was measured using laser-Doppler flowmetry beginning pre-occlusion and persisting through 15 minutes post-reperfusion[[1](#_ENREF_1)](Periflux System 5000; Perimed, Stockholm, Sweden). Intra-operative core temperature was maintained at 37.0±0.1°C using a Digi-Sense temperature controller (Cole-Parmer, Vernon Hills, IL, USA) with a heat-lamp coupled with a thermistor rectal probe model 400 (Yellow Springs Instruments Co., Yellow Springs, OH, USA). Post-operatively, an animal intensive-care-unit (Lyon Electric Company, Chula Vista, CA, USA) was used along with a Thermistemp temperature-controller (Yellow Springs Instruments Co., Yellow Springs, OH, USA) to maintain a temperature of 37°C for a total of 150 minutes following ischemia.

*References*

1. Connolly ES, Jr., Winfree CJ, Stern DM, Solomon RA, Pinsky DJ (1996) Procedural and strain-related variables significantly affect outcome in a murine model of focal cerebral ischemia. Neurosurgery 38: 523-531; discussion 532.
