# Supplementary material for: Complement Inhibition Promotes Endogenous Neurogenesis and Sustained Anti-Inflammatory Neuroprotection following Reperfused Stroke
Source: PLoS One. 2012 Jun 26;7(6):e38664. doi: 10.1371/journal.pone.0038664 (PMC3383680; doi:10.1371/journal.pone.0038664)
Supplement: Methods S2 — Immunohistochemistry Antibodies. (DOCX) [file pone.0038664.s006.docx]

**Methods S2. Immunohistochemistry Antibodies.** Primary antibodies and reagents used for immunohistochemistry include rat anti-BrdU monoclonal antibody(Novus Biologicals, Littleton, CO, USA; 1:100) goat anti-doublecortin(DCx) polyclonal antibody(Santa Cruz Biotechnology, Santa Cruz, CA, USA; 1:50); rabbit anti-glial fibrillary acidic protein(GFAP) polyclonal antibody(Chemicon, Temecula, CA, USA; 1:600); mouse anti-neuronal nuclei(NeuN) monoclonal antibody(Chemicon, Temecula, CA, USA; 1:600); chicken anti-mouse complement C3a receptor(C3aR) antibody(Accurate chemical & scientific corporation, Westbury, NY, USA; 1:200); goat anti-C3aR polyclonal antibody(Santa Cruz Biotechnology, Santa Cruz, CA, USA; 1:200); rat anti-mouse Ly-6G/C monoclonal antibody(BD Pharmingen, San Diego, CA, USA; 1:50); rabbit anti-CD3 polyclonal antibody(Abcam Inc., Cambridge, MA, USA; 1:100) and Neurotrace 640/660 fluorescent Nissl(Molecular Probes, Eugene, OR, USA).  Secondary-antibodies included Alexa Fluor 488 or 594 conjugated anti-mouse IgG, anti-rat IgG, anti-rabbit IgG, anti-chicken IgG and anti-goat IgG(Molecular Probes, Eugene, OR, USA). No significant positive immunostaining was observed when primary antibodies were omitted.
