# Supplementary material for: Complement Inhibition Promotes Endogenous Neurogenesis and Sustained Anti-Inflammatory Neuroprotection following Reperfused Stroke
Source: PLoS One. 2012 Jun 26;7(6):e38664. doi: 10.1371/journal.pone.0038664 (PMC3383680; doi:10.1371/journal.pone.0038664)
Supplement: Methods S3 — Morris Water Maze Procedure. (DOCX) [file pone.0038664.s007.docx]

**Methods S3. Morris Water Maze Procedure[**[**1,2**](#_ENREF_2)**].** Briefly, mice were tested in an 80cm diameter pool filled with opaque water (26°C). The rescue-landing platform (3cm diameter) was submerged 0.5 cm beneath the surface in one of four quadrants. Navigational cues were placed on the walls, and a small flag was placed on the landing platform. The testing began pre-operatively with five consecutive training days, during which three attempts were given to each mouse to find the platform within 120 seconds, with 20 minutes rest between trials. The training sessions occurred at the same time of the day throughout the training and testing periods. On day 4, the flag was removed and the mouse was permitted three attempts to locate the platform using peripheral navigational cues. On day 5, the landing platform was removed and each mouse was placed in the pool for 60 seconds. The time spent in the quadrant where the platform was previously situated is recorded. On post-operative day 7, mice were placed in the same swimming pool without the landing platform for 60 seconds. The time spent in the quadrant where the landing had previously been located was recorded in a masked fashion.

*References*

1. Ten VS, Bradley-Moore M, Gingrich JA, Stark RI, Pinsky DJ (2003) Brain injury and neurofunctional deficit in neonatal mice with hypoxic-ischemic encephalopathy. Behav Brain Res 145: 209-219.

2. Rynkowski MA, Kim GH, Garrett MC, Zacharia BE, Otten ML, et al. (2009) C3a receptor antagonist attenuates brain injury after intracerebral hemorrhage. J Cereb Blood Flow Metab 29: 98-107.
