# Supplementary material for: Complement Inhibition Promotes Endogenous Neurogenesis and Sustained Anti-Inflammatory Neuroprotection following Reperfused Stroke
Source: PLoS One. 2012 Jun 26;7(6):e38664. doi: 10.1371/journal.pone.0038664 (PMC3383680; doi:10.1371/journal.pone.0038664)
Supplement: Methods S4 — Corner-Turn Test Procedure. (DOCX) [file pone.0038664.s008.docx]

**Methods S4. Corner-Turn Test Procedure[**[**1,2**](#_ENREF_3)**].** For this test, the mouse was allowed to walk down a corridor into a 30° corner. The mouse's choice of turn direction for a total of 10 attempts was recorded.

*References*

1. Rynkowski MA, Kim GH, Garrett MC, Zacharia BE, Otten ML, et al. (2009) C3a receptor antagonist attenuates brain injury after intracerebral hemorrhage. J Cereb Blood Flow Metab 29: 98-107.

2. Bouet V, Freret T, Toutain J, Divoux D, Boulouard M, et al. (2007) Sensorimotor and cognitive deficits after transient middle cerebral artery occlusion in the mouse. Exp Neurol 203: 555-567.
